# Supplementary material for: MeFiT: merging and filtering tool for illumina paired-end reads for 16S rRNA amplicon sequencing
Source: BMC Bioinformatics. 2016 Dec 1;17:491. doi: 10.1186/s12859-016-1358-1 (PMC5134250; doi:10.1186/s12859-016-1358-1)
Supplement: Additional file 1: — Supplementary methods, tables and figures. (DOCX 505 kb) [file 12859_2016_1358_MOESM1_ESM.docx]

**Title**

MeFiT: Merging and Filtering Tool for Illumina Paired-End Reads for 16S rRNA Amplicon Sequencing

Hardik I. Parikh^1^ (parikhhi@vcu.edu)

Vishal N. Koparde^2^ (vnkoparde@vcu.edu)

Steven P. Bradley^1^ (bradleysp@vcu.edu)

Gregory A. Buck^1,2^ (gabuck@vcu.edu)

Nihar U. Sheth^2*^ (nsheth@vcu.edu)

*Corresponding Author

^1^Department of Microbiology and Immunology, Virginia Commonwealth University, Richmond, Virginia USA

^2^Center for the Study of Biological Complexity, Virginia Commonwealth University, Richmond, Virginia USA

**Supplementary Information:**

**OTU Clustering and Analysis of Microbial Community**

To demonstrate the utility of MeFiT, we performed taxonomic assignments to characterize a complex microbial community using Mothur^1^. The following section compares the results of OTU-analysis from the high-quality data obtained by classical pipeline implemented in Mothur, to that obtained from MeFiT.

**Dataset**: The 16S rRNA gene sequence data of a mock bacterial community was obtained from the NCBI’s Short Read Archive (SRA) under the BioProject *PRJNA325813*.^2^ The V1-V3 region of the 16S gene was sequenced using version 3 (2 X 300) chemistry on the Illumina MiSeq instrument. The sequences from 12 replicates were combined in order to perform the OTU-analysis. Please refer Ref2 for details on the bacterial composition, genome sizes and 16S gene copy numbers.

**Methods:** The data was analyzed using two different strategies –

1. MothurQC-Mothur: The raw data was analyzed following the standard operating procedure outlined as a part of Mothur MiSeq SOP (http://www.mothur.org/wiki/MiSeq_SOP). The quality control steps included making contigs, aligning sequences, trim sequences, and removing chimeras and non-bacterial sequences. This was followed by OTU clustering at 97% similarity and taxonomic classification using mothur’s implementation of SILVA database.
2. MeFiTQC-Mothur: Quality control steps were performed using MeFiT, with raw overlapping paired-end reads merged into amplicons with default CASPER parameters, followed by filtering the amplicons for quality with *meep* threshold of 1.0. Chimeras and non-bacterial sequences were removed from the high-quality overlapping and non-overlapping amplicons, followed by OTU clustering at 97% similarity and taxonomic classification using mothur’s implementation of SILVA database.

Supplementary File 2 contains the commands executed for both analysis strategies.

**Results**:

The mock community comprised of equal contributions from 19 different bacterial genera – Campylobacter, Salmonella, Escherichia, Megasphaera, Cloacibacillus, Brachyspira, Haemophilus, Staphylococcus, Bacteroides, Faecalibacterium, Streptococcus, Parabacteroides, Oscillibacter, Desulfovibrio, Lactobascillus, Coriobacterium, Oxalobacter, Roseburia, and 1 archaeon Methanobrevibacter. To detect the members of the mock community in the sequence data, we performed OTU clustering at 97% similarity followed by taxonomic classification using Mothur. As seen from table S4, sequences cluster into fewer OTUs with MeFiT high-quality data. In addition, there is significant gain in processing times owing to fewer pairwise comparisons (the distance matrix file size is significantly smaller). Figure S2 shows the identification of all 19 genera in similar relative abundances, with the percent of unclassified data dropping from 8.31% with MothurQC to 5.48% with MeFiTQC. However, the abundances of each genera are not in concordance with their actual contributions in the mock. This is not surprising, as other studies have indicated amplification bias to be a confounder in amplicon-based rRNA gene surveys of microbial communities.^3,4^

**Tables**

**Table S1**: Phylum-level distribution of sequences in SILVA rRNA database (16S, SSU Ref NR, v119) with hyper-variable regions (extracted using V-Xtractor^27^) greater than/equal to 540bp.

| Phylum | 16S Region | | | |
| --- | --- | --- | --- | --- |
|  | V1-V3 | V3-V5 | V4-V6 | V6-V9 |
| Acidobacteria | 0 | 1 | 1 | 1 |
| Actinobacteria | 2 | 3 | 4 | 0 |
| Archaeplastida | 0 | 0 | 0 | 5 |
| Armatimonadetes | 0 | 0 | 0 | 1 |
| BD1-5 | 1 | 0 | 0 | 0 |
| Bacteroidetes | 1 | 11 | 16 | 2 |
| Candidate division BRC1 | 0 | 1 | 1 | 0 |
| Candidate division JS1 | 4 | 0 | 0 | 0 |
| Candidate division OD1 | 4 | 2 | 1 | 4 |
| Candidate division OP11 | 0 | 4 | 19 | 0 |
| Candidate division OP8 | 0 | 0 | 0 | 1 |
| Candidate division SR1 | 0 | 0 | 1 | 0 |
| Candidate division TM7 | 3 | 0 | 1 | 0 |
| Candidate division WS3 | 0 | 1 | 1 | 0 |
| Candidate division WS6 | 0 | 0 | 1 | 0 |
| Chloroflexi | 1 | 0 | 0 | 0 |
| Crenarchaeota | 0 | 0 | 5 | 0 |
| Cyanobacteria | 0 | 1 | 7 | 6 |
| Deferribacteres | 0 | 1 | 0 | 2 |
| Deinococcus-Thermus | 0 | 0 | 1 | 2 |
| Euryarchaeota | 0 | 0 | 33 | 1 |
| Firmicutes | 87 | 32 | 46 | 186 |
| Fusobacteria | 0 | 0 | 1 | 0 |
| Gemmatimonadetes | 0 | 1 | 0 | 0 |
| Nitrospirae | 6 | 0 | 2 | 2 |
| Opisthokonta | 0 | 0 | 0 | 13 |
| Planctomycetes | 0 | 1 | 1 | 2 |
| Proteobacteria | 57 | 66 | 97 | 375 |
| SAR | 0 | 0 | 0 | 2 |
| Spirochaetae | 1 | 0 | 1 | 1 |
| Synergistetes | 0 | 0 | 0 | 1 |
| Thaumarchaeota | 0 | 1 | 5 | 0 |
| Verrucomicrobia | 0 | 0 | 1 | 0 |
| WCHB1-60 | 1 | 0 | 0 | 0 |

**Table S2**: MeFiT sample statistics

| **SampleID** | **Total Reads** | **Overlapping (%)** | **Non Overlapping (%)** | **Quality Filtering Method** | **Threshold** | **Avg Amplicon Length** | **Avg Quality** | **Avg meep** | **HQ Amplicons (%)** | **HQ -  % Overlapping** | **HQ – Avg Amplicon Length** | **HQ - Avg Quality** | **HQ - Avg meep** |
| --- | --- | --- | --- | --- | --- | --- | --- | --- | --- | --- | --- | --- | --- |
| Sample 1 | 46542 | 43102 (92.61%) | 3440 (7.39%) | meep | 1 | 557.55 | 33.93 | 0.63 | 38834 (83.44%) | 99.86 | 553.97 | 35.16 | 0.29 |
| Sample 2 | 46113 | 43140 (93.55%) | 2973 (6.45%) | meep | 1 | 529.52 | 34.14 | 0.57 | 39440 (85.53%) | 99.95 | 524.21 | 35.33 | 0.24 |
| Sample 3 | 35269 | 33719 (95.61%) | 1550 (4.39%) | meep | 1 | 519.6 | 34.77 | 0.42 | 31871 (90.37%) | 99.99 | 515.48 | 35.64 | 0.19 |
| Sample 4 | 26741 | 25179 (94.16%) | 1562 (5.84%) | meep | 1 | 520.65 | 34.55 | 0.48 | 23696 (88.61%) | 99.97 | 515.22 | 35.61 | 0.19 |
| Sample 1 | 46542 | 43102 (92.61%) | 3440 (7.39%) | avgq | 20 | 557.55 | 33.93 | 0.63 | 46373 (99.64%) | 92.94 | 557.38 | 22.99 | 0.61 |
| Sample 2 | 46113 | 43140 (93.55%) | 2973 (6.45%) | avgq | 20 | 529.52 | 34.14 | 0.57 | 45905 (99.55%) | 93.97 | 529.19 | 34.21 | 0.55 |
| Sample 3 | 35269 | 33719 (95.61%) | 1550 (4.39%) | avgq | 20 | 519.6 | 34.77 | 0.42 | 35138 (99.63%) | 95.96 | 519.28 | 34.83 | 0.40 |
| Sample 4 | 26741 | 25179 (94.16%) | 1562 (5.84%) | avgq | 20 | 520.65 | 34.55 | 0.48 | 26617 (99.54%) | 94.59 | 520.27 | 34.63 | 0.46 |

**Table S3:** MeFiT results for simulated dataset

| **Organism** | **V1-V3 length** | **Total Reads** | **Overlapping (%)** | **Non Overlapping (%)** | **Avg Amplicon Length** | **Avg meep** |
| --- | --- | --- | --- | --- | --- | --- |
| *Lactobacillus iners* | 519 | 20000 | 19953 (99.77%) | 47 (0.23 %) | 519.23 | 0.1 |
| *Lactobacillus crispatus* | 512 | 20000 | 19991 (99.95%) | 9 (0.04 %) | 512.05 | 0.09 |
| *Prevotella bivia* | 490 | 20000 | 20000 (100 %) | 0 (0 %) | 490 | 0.06 |
| *Gardnerella vaginalis* | 471 | 20000 | 20000 (100 %) | 0 (0 %) | 471 | 0.05 |
| *Clostridium josui* | 595 | 20000 | 27 (0.14 %) | 19973 (99.86 %) | 614.97 | 0.3 |
| *Campylobacter rectus* | 651 | 20000 | 2 (0.01 %) | 19998 (99.99 %) | 615 | 0.3 |

**Table S4:** OTU analysis of the mock community

| **Analysis** |  | **Total Reads** | **# Amplicons** | **# High-Quality Amplicons** | **OTUs** | **Inverse Simpson Index** | **Distance Matrix File Size (Gb)** |
| --- | --- | --- | --- | --- | --- | --- | --- |
|  |  |  |  |  |  |  |  |
| MothurQC - Mothur |  | 237601 | 237601 | 221379 | 19671 | 12.01 | 18 |
|  |  |  |  |  |  |  |  |
| MeFiTQC - Mothur |  | 237601 | 207037 | 193222 | 1100 | 11.85 | 2.6 |
|  |  |  |  |  |  |  |  |

**Figures**

**Figure S1:** Species-level classification of simulated dataset using STIRRUPS^27^. Classification on a set of only overlapping high-quality reads result in community composed of four species with shorter V1-V3 amplicons (*G. vaginalis, L. crispatus, L. iners, P. bivia*). However, including both overlapping and non-overlapping high-quality reads result in the identification of community comprising of all six species (in addition, *C. rectus and C. josui*), with more accurate relative abundances.


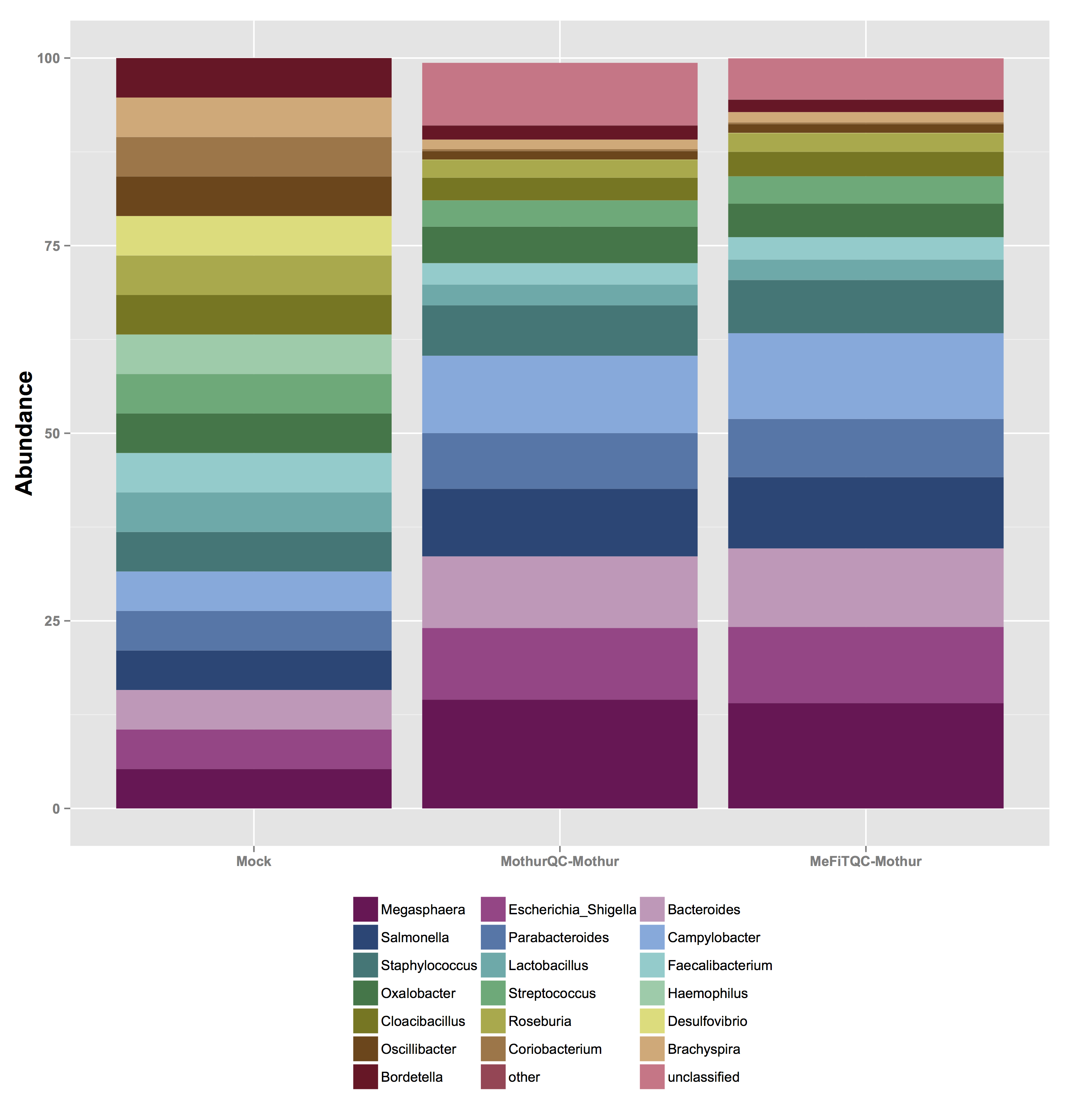


**Figure S2:** Relative abundance of bacterial genera in mock community

**Reference:**

1. Schloss PD, Westcott SL, Ryabin T, HAll JR, Hartmann M, Hollister EB, Lesniewski RA, Oakley BB, Parks DH, Robinson CJ, Sahl JW, Stress B, Thallinger GG, Van Horn DJ, Weber CF. Introducing mothur: open-source, platform-independent, community-supported software for describing and comparing microbial communities. Appl Environ Microbiol. 2009;75:7537-41.
2. Allen HK, Bayles DO, Looft T, Trachsel J, Bass BE, Alt DP, Bearson SMD, Nicholson T and Casey TA. Pipeline for amplifying and analyzing amplicons of the V1-V3 region of the 16S rRNA gene. BMC Res Notes, 2016;9:380.
3. Kennedy K, Hall MW, Lynch MD, Moreno-Hagelsieb G, Neufeld JD. Evaluating bias of Illumina-based bacterial 16S rRNA gene profiles. Appl Environ Microbiol. 2014;80(18):5717–5722.
4. Brooks JP, Edwards DJ, Harwich MD, Rivera MC, Fettweis JM, Serrano MG, Reris RA, Sheth NU, Huang B, Girerd P. The truth about metagenomics: quantifying and counteracting bias in 16S rRNA studies. BMC Microbiol. 2015;15(1):66.
